# (2,6-Bis{5-amino-3-*tert*-butyl-4-[(3-methyl-1,2,4-thia­diazol-5-yl)diazen­yl]-1*H*-pyrazol-1-yl}-4-oxo-1,4-dihydro-1,3,5-triazin-1-ido)methanol(phenol)sodium phenol tetra­solvate

**DOI:** 10.1107/S160053681001086X

**Published:** 2010-03-27

**Authors:** Hiroki Shibata, Jin Mizuguchi

**Affiliations:** a Department of Applied Physics, Graduate School of Engineering, Yokohama National University, Tokiwadai 79-5, Hodogaya-ku, Yokohama 240-8501, Japan

## Abstract

The title compound, [Na(C_23_H_28_N_17_OS_2_)(CH_3_OH)(C_6_H_5_OH)]·4C_6_H_5_OH, is a five-coordinate Na^I^ complex. The Na^+^ cation is bound to three N atoms of the triazinide ligand, two from each pyrazole ring and one from the central deprotonated triazine ring system. O atoms from a methanol and a phenol mol­ecule complete the five-coordinate NaN_3_O_2_ coordination environment. The asymmetric unit also includes three complete and two half phenol mol­ecules, four of which are hydrogen bonded to the N atoms of the thia­diazole ring. Two of the phenol solvent mol­ecules are disordered over two discrete inversion centres. The triazinide ligand is essentially planar (mean deviation from the least-squares plane = 0.0524 Å), with the methyl groups of the *tert*-butyl substituents on the pyrazole rings located above and below the plane. The planarity of this system is further assisted by the formation of four intra­molecular N—H⋯N hydrogen bonds between the N—H bonds of both amino groups on the pyrazole rings and the N atoms of the triazine ring and also from each of the adjacent diazene (–N=N–) bonds. The highly polar mol­ecules are stacked along the *a* axis through the central Na atom sandwiched by two kinds of alternating inter­molecular hydrogen bonds: O(carbon­yl)⋯H—O(methanol)/Na/O—H(phenol)⋯O(carbon­yl). These inter­actions form two polymer chains per mol­ecule.

## Related literature

For details of azo pigments, see: Herbst & Hunger (2004 ▶). For the synthesis of the title compound, see: Nagata & Tateishi (2009 ▶). For the structures of other azo complexes with five-coordinate Na^+^, see: Mizuguchi *et al.* (2007 ▶); Sato, Shibata *et al.* (2008 ▶); Sato, Uta *et al.* (2008 ▶). For the structure of a related ligand, see: Shibata & Mizuguchi (2010 ▶).
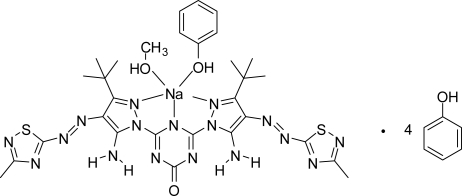


## Experimental

### 

#### Crystal data

[Na(C_23_H_28_N_17_OS_2_)(CH_4_O)(C_6_H_6_O)]·4C_6_H_6_O
*M* *_r_* = 1148.30
Triclinic, 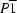

*a* = 8.38964 (15) Å
*b* = 18.8780 (3) Å
*c* = 20.4060 (4) Å
α = 114.103 (1)°
β = 96.580 (1)°
γ = 95.650 (1)°
*V* = 2892.83 (9) Å^3^
*Z* = 2
Cu *K*α radiation
μ = 1.46 mm^−1^
*T* = 93 K
0.50 × 0.30 × 0.20 mm

#### Data collection

Rigaku R-AXIS RAPID diffractometer
Absorption correction: multi-scan (*ABSCOR*; Higashi, 1995 ▶) *T*_min_ = 0.345, *T*_max_ = 0.748
32979 measured reflections
10340 independent reflections
7267 reflections with *I* > 2σ(*I* ^2^)
*R*_int_ = 0.054

#### Refinement

*R*[*F* ^2^ > 2σ(*F* ^2^)] = 0.064
*wR*(*F* ^2^) = 0.196
*S* = 0.99
10340 reflections
819 parameters
156 restraints
H-atom parameters constrained
Δρ_max_ = 0.66 e Å^−3^
Δρ_min_ = −0.47 e Å^−3^

### 

Data collection: *PROCESS-AUTO* (Rigaku, 1998 ▶); cell refinement: *PROCESS-AUTO*; data reduction: *CrystalStructure* (Rigaku/MSC & Rigaku, 2006 ▶); program(s) used to solve structure: *SIR2004* (Burla *et al.*, 2005 ▶); program(s) used to refine structure: *SHELXL97* (Sheldrick, 2008 ▶); molecular graphics: *ORTEPIII* (Burnett & Johnson, 1996 ▶); software used to prepare material for publication: *CrystalStructure* (Rigaku/MSC & Rigaku, 2006 ▶).

## Supplementary Material

Crystal structure: contains datablocks global, I. DOI: 10.1107/S160053681001086X/sj2750sup1.cif

Structure factors: contains datablocks I. DOI: 10.1107/S160053681001086X/sj2750Isup2.hkl

Additional supplementary materials:  crystallographic information; 3D view; checkCIF report

## Figures and Tables

**Table:** 

Na1—O8	2.336 (3)
Na1—N7	2.420 (3)
Na1—O2	2.437 (2)
Na1—N11	2.642 (3)
Na1—N5	2.644 (3)
N3—N4	1.295 (4)
N12—N13	1.308 (3)

**Table:** 

N7—Na1—N11	65.51 (8)
N7—Na1—N5	65.04 (8)

**Table 2:** Hydrogen-bond geometry (Å, °)

*D*—H⋯*A*	*D*—H	H⋯*A*	*D*⋯*A*	*D*—H⋯*A*
O2—H2*O*⋯O1^i^	0.84	1.84	2.682 (2)	175
O3—H3*O*⋯N1	0.84	2.01	2.835 (3)	166
O4—H4*O*⋯N14	0.84	1.99	2.757 (3)	152
O5—H5*O*⋯N2	0.84	2.10	2.926 (6)	170
O5*A*—H5*OA*⋯N2	0.84	2.02	2.831 (14)	162
O7—H7*O*⋯N15	0.84	2.21	2.975 (5)	152
O8—H8*O*⋯O1^ii^	0.84	1.99	2.822 (3)	173
N16—H16*M*⋯N3	0.88	2.16	2.719 (4)	121
N16—H16*N*⋯N9	0.88	2.05	2.660 (3)	126
N17—H17*M*⋯N13	0.88	2.13	2.705 (2)	122
N17—H17*N*⋯N8	0.88	2.06	2.661 (3)	125

Symmetry codes: (i) 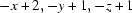; (ii) 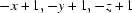.

**Fig. 1.**
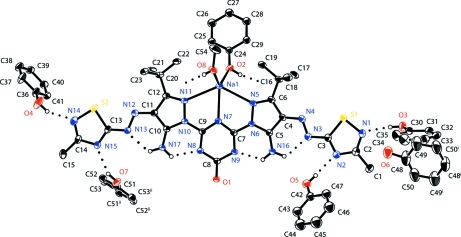
ORTEP plot of I. Hydrogen atoms except for those involved in hydrogen bonds (dotted lines) are omitted for clarity. Symmetry codes: (i) -x+1, -y+2, -z+1 and (ii) -x-1, -y-1, -z.

**Fig. 2.**
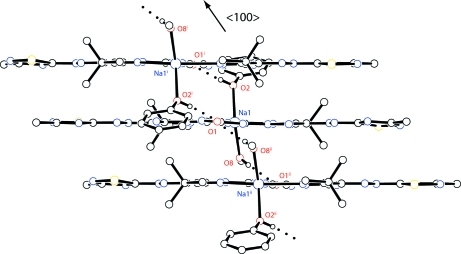
The molecular stack along <100>. Symmetry codes: (i) -x, -y, -z and (ii) -x-1, -y,-z.

## supplementary crystallographic information

### Comment

Azo pigments are widely used in imaging and painting industries because of their versatile colors, high tinctorial strength as well as their low price (Herbst & Hunger, 2004). Some novel azo pigments have recently been reported by Nagata *et al.* (2009) which include 2,6-bis[5-amino-3-*tert*-butyl-4-(3-methyl-1,2,4-thiadiazol-5-yldiazenyl)-*1H*-pyrazol-1-yl]-1,3,5-triazin-4(1*H*)-one (*i.e.* Na-free compound of the title compound with one additional H atom at the N atom of the triazine one). In the course of our structural studies on this compound, however, we have isolated two kinds of single crystals from the reaction product: bis-azo and mono-azo compounds. To our surprise, the former is found to include a Na^I^-atom as the central metal (*i.e.* the title compound); whereas the latter is a Na^I^-free mono-azo compound. In the synthesis of azo pigments, it is quite common to use sodium nitrite for the preparation of diazonium salts. However, the inclusion of Na in the final product has not been reported to date. This paper deals with the structure of the Na^I^-containing bis-azo compound, while the Na^I^-free mono-azo compound will be reported elsewhere (Shibata & Mizuguchi, 2010).

The title compound, C_30_H_38_N_17_NaO_3_S_2_^.^4(C_6_H_6_O), is a five-coordinate Na-bisazo complex, comprising as ligands two N atoms of each pyrazol ring and one N atom of the central triazine one as well as two O atoms of the solvent molecules: one methanol and one phenol. Fig. 1 shows the *ORTEP* plot of I. The asymmetric unit also includes three full phenol molecules (C30—C35/O3, C36—C41/O4, and C42—C47/O5) and two half molecules (C48—C50/O6 and C51—C53/O7). In four of these molecules, the O—H group of the molecule is hydrogen-bonded to the N atom of the 1,2,4-thiadiazol ring (N1, N2, N14, and N15) through O—H···N hydrogen bonds. On the other hand, the fifth phenol molecule (C48—C50/O6) remains free. There are four N—H···N intramolecular hydrogen bonds formed between the N—H of the amino group of the pyrazol ring and the N atom of the azo bond: N16—H16M···N3, N16—H16N···N9, N17—H17M···N13, and N17—H17N···N8. It is remarkable to note that the central Na atom bridges two monoazo moieties in a *cis* fashion to make the molecule entirely flat (mean deviation from the least-squares plane with a methyl group from the *t*-butyl substituent on the pyrazol ring above and below that plane: 0.0524 Å). Additionally, the four intramolecular N—H···N hydrogen bonds also contribute to the planarity of the system. The occurrence of the *cis* form is quite unusual, because the *trans* form of the Na-free structure as formed by free rotation around C7—N6 or C9—N10 is more stable according to molecular orbital calculations. However, the Na-coordination appears here to be the driving force to form the *cis* structure.

The incorporation of the Na^I^ cation to form a complex is due largely to the formation of strong Na—N bonds as shown by the distances Na1—N5 = 2.644 (3), Na1—N7 = 2.420 (3), and Na1—N11 = 2.642 (3) Å. The Na1—N5 and Na1—N11 distances are nearly equal, as indicated by the fact that the angles N5–Na1–N7 and N7–Na1–N11 are closely similar at  65.04 (8) and 65.51 (8)°, respectively. Furthermore, the O atoms of a phenol and methanol molecule are also coordinated to the Na atom, Na1—O2 = 2.437 (2) and Na1—O8 = 2.336 (3) Å, respectively generating a five-coordinate Na^I^-bis-azo complex. Similar five-coordinate Na^I^-complexes are found in other azo compounds (Mizuguchi *et al.*, 2007; Sato, Shibata *et al.*, 2008; Sato, Uta *et al.*, 2008). The azo bond in this molecule is also noteworthy. The bond lengths, N3—N4 and N12—N13 are 1.295 (4) and 1.308 (3) Å, respectively. These are longer than the standard N=N bond of about 1.24 Å, found in other bis-azo compounds (Mizuguchi *et al.*, 2007;Sato, Uta *et al.*, 2008).

Fig. 2 illustrates the molecular stack along the *a* axis. The methanol and phenol ligands to the Na atom are positioned above and below the bis-azo skeleton. The polar bis-azo molecules are arranged alternately in such a way as to effectively cancel their dipole moments to lower the lattice energy. In addition, two stacked columns are formed composed of the central Na^I^ atom sandwiched by alternating two kinds of intermolecular hydrogen bonds: O (carbonyl oxygen: O1^ii^)···O—H (methanol: O8)/Na1/O—H (phenol: O2)···O (carbonyl oxygen: O1^i^)(Fig. 2). This forms two one-dimensional polymer-chains per molecule and assures a high thermal-stability of the compound.

### Experimental

The title compound was synthesized as described by Nagata *et al.* (2009). The structure reported here is of the Na^I^ complex product which made up approximately 80% of the product mixture according by emission spectrochemical analysis. A single crystal suitable for X-ray analysis was grown from a mixed solvent of methanol and phenol (1:1 in molar ratio) prepared at 80 °C. Needle shaped crystals were obtained in a closed system saturated with methanol vapor after standing for one week.

### Refinement

The C atoms of the phenol molecule which acts as a ligand to the Na atom were disordered over two positions (C24—C29 and C24A—C29A) with occupancies of 0.786 (6)/0.214 (6), respectively. The entire molecule of another phenol was disordered over two sites (C42—C47/O5 and C42A—C47A/O5A) with occupancies of 0.750 (5) and 0.250 (5), respectively. The benzene rings of these disordered phenol molecules were refined anisotropically as rigid groups. The two half phenol-molecules were disordered about a center of symmetry (C48—C50/O6 and C51—C53/O7) with an occupancy of 0.5. Because of this disorder, there is a discrepancy between the H atom counts in the_chemical_formula_sum and the formula from the_atom_site data: one H atom is missing. The occupancies of all disorders described above extend to the associated H atoms. All H atoms were placed in geometrically idealized position and constrained to ride on their parent atoms, with C—H = 0.95, and 0.98 Å, and *U*_iso_(H) = 1.2 and 1.5 *U*_eq_(C), respectively, and with O—H = 0.84, and N—H = 0.88Å and *U*_iso_(H) = 1.2.

### Crystal data

**Table:**

[Na(C_23_H_28_N_17_OS_2_)(CH_4_O)(C_6_H_6_O)]·4C_6_H_6_O	*Z* = 2
*M**_r_* = 1148.30	*F*(000) = 1208.00
Triclinic, *P*1	*D*_x_ = 1.318 Mg m^−^^3^
Hall symbol: -P 1	Cu *K*α radiation, λ = 1.54187 Å
*a* = 8.38964 (15) Å	Cell parameters from 26974 reflections
*b* = 18.8780 (3) Å	θ = 4.2–68.2°
*c* = 20.4060 (4) Å	µ = 1.46 mm^−^^1^
α = 114.103 (1)°	*T* = 93 K
β = 96.580 (1)°	Needle, yellow
γ = 95.650 (1)°	0.50 × 0.30 × 0.20 mm
*V* = 2892.83 (9) Å^3^	

### Data collection

**Table:**

Rigaku R-AXIS RAPID diffractometer	7267 reflections with *F*^2^ > 2σ(*F*^2^)
ω scans	*R*_int_ = 0.054
Absorption correction: multi-scan (*ABSCOR*; Higashi, 1995)	θ_max_ = 68.2°
*T*_min_ = 0.345, *T*_max_ = 0.748	*h* = −9→9
32979 measured reflections	*k* = −20→22
10340 independent reflections	*l* = −24→24

### Refinement

**Table:**

Refinement on *F*^2^	156 restraints
*R*[*F*^2^ > 2σ(*F*^2^)] = 0.064	H-atom parameters constrained
*wR*(*F*^2^) = 0.196	*w* = 1/[σ^2^(*F*_o_^2^) + (0.101*P*)^2^ + 2.2948*P*] where *P* = (*F*_o_^2^ + 2*F*_c_^2^)/3
*S* = 0.99	(Δ/σ)_max_ = 0.001
10340 reflections	Δρ_max_ = 0.66 e Å^−^^3^
819 parameters	Δρ_min_ = −0.47 e Å^−^^3^

### Special details

**Table:**

Geometry. All esds (except the esd in the dihedral angle between two l.s. planes) are estimated using the full covariance matrix. The cell esds are taken into account individually in the estimation of esds in distances, angles and torsion angles; correlations between esds in cell parameters are only used when they are defined by crystal symmetry. An approximate (isotropic) treatment of cell esds is used for estimating esds involving l.s. planes.
Refinement. Refinement of *F*^2^ against ALL reflections. The weighted *R*-factor wR and goodness of fit *S* are based on *F*^2^, conventional *R*-factors *R* are based on *F*, with *F* set to zero for negative *F*^2^. The threshold expression of *F*^2^ > σ(*F*^2^) is used only for calculating *R*-factors(gt) etc. and is not relevant to the choice of reflections for refinement. *R*-factors based on *F*^2^ are statistically about twice as large as those based on *F*, and *R*- factors based on ALL data will be even larger.

### Fractional atomic coordinates and isotropic or equivalent isotropic displacement parameters (Å^2^)

**Table:**

	*x*	*y*	*z*	*U*_iso_*/*U*_eq_	Occ. (<1)
S1	1.47119 (10)	0.92371 (4)	0.62076 (4)	0.0412 (2)	
S2	−0.01553 (10)	0.09004 (5)	0.26699 (4)	0.0442 (2)	
Na1	0.70228 (15)	0.50735 (7)	0.35073 (6)	0.0382 (3)	
O1	0.8097 (3)	0.48489 (11)	0.65787 (10)	0.0342 (5)	
O2	0.8888 (3)	0.45383 (13)	0.26640 (11)	0.0428 (5)	
H2O	0.9806	0.4741	0.2926	0.051*	
O3	1.8364 (3)	1.11563 (15)	0.72119 (14)	0.0583 (7)	
H3O	1.7553	1.0805	0.7099	0.070*	
O4	−0.3653 (3)	−0.08841 (19)	0.18027 (15)	0.0843 (11)	
H4O	−0.2885	−0.0683	0.2158	0.101*	
O6	1.2701 (9)	1.3726 (4)	0.9187 (4)	0.104 (2)	0.50
H6O	1.2690	1.3365	0.9328	0.124*	0.50
O7	0.3015 (6)	0.1073 (2)	0.5196 (2)	0.0435 (11)	0.50
H7O	0.2190	0.0895	0.4867	0.052*	0.50
O8	0.4989 (3)	0.57323 (15)	0.32562 (14)	0.0543 (7)	
H8O	0.4084	0.5590	0.3341	0.065*	
N1	1.5988 (3)	0.98531 (15)	0.69533 (16)	0.0435 (7)	
N2	1.4734 (3)	0.89848 (15)	0.73354 (15)	0.0423 (6)	
N3	1.2860 (3)	0.80306 (14)	0.63605 (14)	0.0389 (6)	
N4	1.2247 (3)	0.78470 (14)	0.56894 (14)	0.0367 (6)	
N5	0.9211 (3)	0.62032 (14)	0.45304 (13)	0.0359 (6)	
N6	0.9431 (3)	0.61113 (13)	0.51868 (13)	0.0318 (6)	
N7	0.7394 (3)	0.50109 (14)	0.46751 (13)	0.0322 (6)	
N8	0.6688 (3)	0.43844 (14)	0.54359 (13)	0.0329 (6)	
N9	0.8837 (3)	0.54727 (14)	0.58893 (13)	0.0321 (6)	
N10	0.5377 (3)	0.39052 (14)	0.42586 (13)	0.0333 (6)	
N11	0.5108 (3)	0.39071 (15)	0.35605 (13)	0.0349 (6)	
N12	0.2410 (3)	0.22050 (14)	0.33247 (13)	0.0354 (6)	
N13	0.2218 (3)	0.19251 (14)	0.38056 (13)	0.0356 (6)	
N14	−0.1125 (4)	0.01872 (16)	0.28168 (16)	0.0488 (7)	
N15	0.0696 (3)	0.08488 (14)	0.38837 (14)	0.0373 (6)	
N16	1.1016 (3)	0.67754 (15)	0.63825 (13)	0.0378 (6)	
H16N	1.0588	0.6422	0.6517	0.045*	
H16M	1.1748	0.7174	0.6692	0.045*	
N17	0.4406 (3)	0.31137 (15)	0.48461 (13)	0.0369 (6)	
H17N	0.5025	0.3423	0.5266	0.044*	
H17M	0.3762	0.2695	0.4805	0.044*	
C1	1.6865 (5)	1.0074 (2)	0.8222 (2)	0.0545 (9)	
H1A	1.7624	1.0501	0.8219	0.082*	
H1B	1.7478	0.9715	0.8344	0.082*	
H1C	1.6169	1.0293	0.8586	0.082*	
C2	1.5832 (4)	0.96327 (19)	0.74835 (19)	0.0444 (8)	
C3	1.4027 (4)	0.87033 (17)	0.66531 (18)	0.0386 (7)	
C4	1.1104 (4)	0.71951 (16)	0.53837 (16)	0.0345 (7)	
C5	1.0559 (4)	0.67071 (16)	0.57146 (16)	0.0320 (7)	
C6	1.0198 (4)	0.68461 (16)	0.46545 (17)	0.0361 (7)	
C7	0.8499 (4)	0.54981 (16)	0.52534 (16)	0.0310 (6)	
C8	0.7878 (4)	0.49012 (16)	0.59835 (16)	0.0314 (6)	
C9	0.6561 (4)	0.44673 (17)	0.48198 (15)	0.0310 (6)	
C10	0.4443 (4)	0.32788 (17)	0.42756 (16)	0.0333 (7)	
C11	0.3520 (4)	0.28622 (17)	0.35716 (16)	0.0349 (7)	
C12	0.3997 (4)	0.32902 (18)	0.31600 (16)	0.0353 (7)	
C13	0.1050 (4)	0.12619 (17)	0.35169 (16)	0.0346 (7)	
C14	−0.0544 (4)	0.02450 (18)	0.34631 (18)	0.0400 (7)	
C15	−0.1193 (5)	−0.0316 (2)	0.3746 (2)	0.0545 (9)	
H15A	−0.2214	−0.0631	0.3432	0.082*	
H15B	−0.1393	−0.0023	0.4243	0.082*	
H15C	−0.0401	−0.0663	0.3750	0.082*	
C16	1.0319 (5)	0.71468 (17)	0.40775 (17)	0.0436 (8)	
C17	1.2056 (5)	0.7151 (2)	0.3907 (2)	0.0552 (10)	
H17A	1.2826	0.7492	0.4351	0.083*	
H17B	1.2140	0.7348	0.3534	0.083*	
H17C	1.2310	0.6615	0.3727	0.083*	
C18	0.9917 (6)	0.79872 (19)	0.4363 (2)	0.0580 (10)	
H18A	0.8826	0.7986	0.4490	0.087*	
H18B	0.9957	0.8179	0.3984	0.087*	
H18C	1.0713	0.8332	0.4796	0.087*	
C19	0.9102 (5)	0.6628 (2)	0.33927 (19)	0.0579 (11)	
H19A	0.9341	0.6090	0.3205	0.087*	
H19B	0.9181	0.6830	0.3023	0.087*	
H19C	0.8000	0.6631	0.3508	0.087*	
C20	0.3341 (4)	0.30941 (19)	0.23735 (16)	0.0389 (7)	
C21	0.3634 (6)	0.2270 (2)	0.18819 (19)	0.0658 (12)	
H21A	0.4805	0.2268	0.1893	0.099*	
H21B	0.3097	0.2122	0.1381	0.099*	
H21C	0.3189	0.1893	0.2057	0.099*	
C22	0.4140 (5)	0.3709 (2)	0.21546 (19)	0.0547 (10)	
H22A	0.3912	0.4229	0.2468	0.082*	
H22B	0.3703	0.3579	0.1646	0.082*	
H22C	0.5317	0.3714	0.2210	0.082*	
C23	0.1522 (4)	0.3110 (2)	0.22894 (17)	0.0441 (8)	
H23A	0.0976	0.2701	0.2403	0.066*	
H23B	0.1101	0.3013	0.1787	0.066*	
H23C	0.1317	0.3625	0.2624	0.066*	
C24	0.9025 (5)	0.43178 (19)	0.19536 (13)	0.0392 (13)	0.786 (6)
C25	0.8044 (4)	0.36536 (19)	0.14096 (16)	0.0517 (13)	0.786 (6)
H25	0.7270	0.3347	0.1533	0.062*	0.786 (6)
C26	0.8195 (4)	0.34377 (19)	0.06846 (14)	0.0626 (15)	0.786 (6)
H26	0.7525	0.2984	0.0313	0.075*	0.786 (6)
C27	0.9327 (5)	0.3886 (2)	0.05035 (16)	0.0588 (15)	0.786 (6)
H27	0.9431	0.3739	0.0008	0.071*	0.786 (6)
C28	1.0308 (5)	0.4550 (2)	0.1048 (2)	0.0534 (15)	0.786 (6)
H28	1.1081	0.4857	0.0924	0.064*	0.786 (6)
C29	1.0156 (5)	0.47662 (17)	0.1773 (2)	0.0456 (14)	0.786 (6)
H29	1.0827	0.5220	0.2144	0.055*	0.786 (6)
C24A	0.9176 (15)	0.4523 (8)	0.1933 (5)	0.033 (4)	0.214 (6)
C25A	0.7821 (11)	0.4243 (8)	0.1384 (5)	0.045 (4)	0.214 (6)
H25A	0.6774	0.4145	0.1493	0.054*	0.214 (6)
C26A	0.7998 (12)	0.4108 (7)	0.0676 (5)	0.050 (4)	0.214 (6)
H26A	0.7072	0.3917	0.0300	0.060*	0.214 (6)
C27A	0.9531 (15)	0.4252 (7)	0.0516 (6)	0.044 (4)	0.214 (6)
H27A	0.9652	0.4160	0.0032	0.053*	0.214 (6)
C28A	1.0885 (11)	0.4532 (8)	0.1065 (8)	0.037 (4)	0.214 (6)
H28A	1.1933	0.4631	0.0956	0.044*	0.214 (6)
C29A	1.0708 (12)	0.4667 (8)	0.1773 (7)	0.032 (4)	0.214 (6)
H29A	1.1634	0.4858	0.2148	0.039*	0.214 (6)
C30	1.7987 (5)	1.1878 (2)	0.7660 (2)	0.0549 (10)	
C31	1.9181 (6)	1.2527 (2)	0.7902 (2)	0.0623 (11)	
H31	2.0216	1.2477	0.7756	0.075*	
C32	1.8840 (7)	1.3265 (2)	0.8368 (2)	0.0718 (14)	
H32	1.9667	1.3712	0.8550	0.086*	
C33	1.7335 (8)	1.3350 (3)	0.8566 (3)	0.0808 (15)	
H33	1.7113	1.3853	0.8871	0.097*	
C34	1.6159 (7)	1.2702 (3)	0.8321 (3)	0.0838 (15)	
H34	1.5117	1.2755	0.8458	0.101*	
C35	1.6479 (6)	1.1958 (3)	0.7868 (3)	0.0762 (13)	
H35	1.5659	1.1510	0.7703	0.091*	
C36	−0.3128 (4)	−0.1420 (2)	0.1217 (2)	0.0550 (10)	
C37	−0.4253 (5)	−0.1824 (3)	0.0581 (2)	0.0708 (13)	
H37	−0.5345	−0.1729	0.0567	0.085*	
C38	−0.3786 (5)	−0.2362 (2)	−0.0028 (2)	0.0577 (10)	
H38	−0.4548	−0.2624	−0.0467	0.069*	
C39	−0.2229 (5)	−0.2522 (2)	−0.00060 (18)	0.0497 (9)	
H39	−0.1909	−0.2896	−0.0426	0.060*	
C40	−0.1127 (5)	−0.2129 (2)	0.06386 (18)	0.0479 (8)	
H40	−0.0050	−0.2242	0.0661	0.057*	
C41	−0.1577 (4)	−0.15748 (19)	0.12469 (18)	0.0435 (8)	
H41	−0.0810	−0.1303	0.1684	0.052*	
O5	1.2733 (5)	0.8315 (3)	0.8091 (3)	0.0608 (12)	0.750 (5)
H5O	1.3243	0.8554	0.7891	0.073*	0.750 (5)
C42	1.2431 (6)	0.8841 (2)	0.87364 (18)	0.0589 (16)	0.750 (5)
C43	1.1592 (6)	0.85495 (18)	0.9146 (2)	0.0800 (19)	0.750 (5)
H43	1.1281	0.7998	0.8978	0.096*	0.750 (5)
C44	1.1207 (7)	0.9064 (3)	0.9800 (2)	0.107 (2)	0.750 (5)
H44	1.0633	0.8864	1.0080	0.128*	0.750 (5)
C45	1.1662 (7)	0.9870 (2)	1.0046 (2)	0.115 (3)	0.750 (5)
H45	1.1399	1.0221	1.0493	0.138*	0.750 (5)
C46	1.2501 (7)	1.01619 (17)	0.9636 (2)	0.103 (2)	0.750 (5)
H46	1.2812	1.0713	0.9804	0.123*	0.750 (5)
C47	1.2886 (5)	0.9648 (2)	0.8982 (2)	0.0742 (17)	0.750 (5)
H47	1.3460	0.9847	0.8702	0.089*	0.750 (5)
O5A	1.2908 (14)	0.7925 (7)	0.7733 (6)	0.046 (3)	0.250 (5)
H5OA	1.3261	0.8288	0.7624	0.055*	0.250 (5)
C42A	1.2674 (13)	0.8238 (6)	0.8441 (4)	0.042 (3)	0.250 (5)
C43A	1.2089 (12)	0.7696 (5)	0.8690 (4)	0.044 (3)	0.250 (5)
H43A	1.1923	0.7149	0.8380	0.053*	0.250 (5)
C44A	1.1747 (12)	0.7953 (6)	0.9394 (5)	0.059 (4)	0.250 (5)
H44A	1.1347	0.7583	0.9564	0.070*	0.250 (5)
C45A	1.1989 (14)	0.8753 (7)	0.9848 (4)	0.072 (4)	0.250 (5)
H45A	1.1755	0.8929	1.0329	0.086*	0.250 (5)
C46A	1.2575 (14)	0.9295 (5)	0.9598 (5)	0.073 (4)	0.250 (5)
H46A	1.2741	0.9841	0.9909	0.088*	0.250 (5)
C47A	1.2917 (14)	0.9038 (5)	0.8895 (6)	0.059 (4)	0.250 (5)
H47A	1.3317	0.9408	0.8724	0.071*	0.250 (5)
C48	1.3808 (7)	1.4337 (3)	0.9641 (5)	0.107 (2)	
C49	1.3945 (9)	1.4900 (4)	0.9377 (4)	0.123 (2)	
H49	1.3234	1.4847	0.8957	0.148*	
C50	1.4826 (9)	1.4444 (4)	1.0249 (4)	0.119 (2)	
H50	1.4705	1.4057	1.0433	0.143*	
C51	0.3909 (5)	0.0526 (2)	0.5112 (2)	0.0512 (9)	
C52	0.3499 (5)	−0.0221 (2)	0.4556 (2)	0.0505 (9)	
H52	0.2458	−0.0376	0.4256	0.061*	
C53	0.4588 (5)	−0.0747 (2)	0.4431 (2)	0.0511 (9)	
H53	0.4315	−0.1254	0.4036	0.061*	
C54	0.4742 (6)	0.5906 (4)	0.2625 (3)	0.0928 (18)	
H54A	0.5798	0.6023	0.2497	0.139*	
H54B	0.4180	0.6363	0.2738	0.139*	
H54C	0.4083	0.5451	0.2213	0.139*	

### Atomic displacement parameters (Å^2^)

**Table:**

	*U*^11^	*U*^22^	*U*^33^	*U*^12^	*U*^13^	*U*^23^
S1	0.0477 (5)	0.0303 (4)	0.0446 (5)	0.0054 (3)	0.0105 (4)	0.0144 (3)
S2	0.0453 (5)	0.0398 (4)	0.0356 (4)	0.0109 (4)	−0.0028 (3)	0.0058 (3)
Na1	0.0423 (7)	0.0411 (7)	0.0361 (7)	0.0094 (5)	0.0071 (5)	0.0206 (5)
O1	0.0411 (12)	0.0334 (11)	0.0301 (11)	0.0062 (9)	0.0051 (9)	0.0157 (9)
O2	0.0402 (13)	0.0472 (13)	0.0325 (12)	0.0089 (10)	0.0005 (9)	0.0094 (10)
O3	0.0552 (16)	0.0481 (14)	0.0577 (16)	−0.0048 (12)	0.0098 (13)	0.0116 (12)
O4	0.0449 (16)	0.095 (2)	0.0560 (17)	0.0093 (15)	0.0047 (13)	−0.0235 (16)
O6	0.094 (5)	0.077 (5)	0.149 (7)	−0.007 (4)	−0.009 (5)	0.070 (5)
O7	0.047 (3)	0.040 (2)	0.037 (2)	0.008 (2)	−0.005 (2)	0.012 (2)
O8	0.0424 (14)	0.0757 (17)	0.0714 (17)	0.0151 (12)	0.0148 (12)	0.0550 (15)
N1	0.0406 (16)	0.0363 (14)	0.0507 (17)	0.0072 (12)	0.0099 (13)	0.0147 (13)
N2	0.0465 (17)	0.0359 (14)	0.0449 (16)	0.0109 (12)	0.0066 (13)	0.0169 (13)
N3	0.0461 (17)	0.0299 (13)	0.0410 (15)	0.0116 (12)	0.0108 (12)	0.0134 (12)
N4	0.0473 (16)	0.0243 (12)	0.0393 (15)	0.0108 (11)	0.0133 (12)	0.0119 (11)
N5	0.0536 (17)	0.0288 (13)	0.0306 (13)	0.0106 (12)	0.0109 (12)	0.0159 (11)
N6	0.0426 (15)	0.0285 (12)	0.0279 (13)	0.0064 (11)	0.0068 (11)	0.0152 (10)
N7	0.0392 (15)	0.0299 (13)	0.0307 (13)	0.0095 (11)	0.0081 (11)	0.0146 (11)
N8	0.0370 (14)	0.0331 (13)	0.0288 (13)	0.0060 (11)	0.0048 (11)	0.0135 (11)
N9	0.0390 (15)	0.0301 (13)	0.0301 (13)	0.0061 (11)	0.0059 (11)	0.0157 (11)
N10	0.0374 (15)	0.0373 (14)	0.0267 (13)	0.0049 (11)	0.0024 (10)	0.0162 (11)
N11	0.0380 (15)	0.0417 (14)	0.0291 (13)	0.0112 (12)	0.0061 (11)	0.0179 (12)
N12	0.0389 (15)	0.0342 (13)	0.0340 (14)	0.0131 (11)	0.0088 (11)	0.0132 (11)
N13	0.0374 (15)	0.0336 (13)	0.0321 (13)	0.0069 (11)	0.0039 (11)	0.0106 (11)
N14	0.0481 (18)	0.0370 (15)	0.0480 (17)	0.0084 (13)	0.0013 (14)	0.0062 (13)
N15	0.0391 (15)	0.0313 (13)	0.0366 (14)	0.0071 (11)	0.0075 (11)	0.0091 (11)
N16	0.0476 (16)	0.0338 (14)	0.0327 (14)	0.0035 (12)	0.0073 (12)	0.0153 (11)
N17	0.0428 (16)	0.0368 (14)	0.0310 (13)	−0.0010 (12)	0.0028 (11)	0.0168 (11)
C1	0.047 (2)	0.058 (2)	0.049 (2)	0.0053 (18)	0.0004 (17)	0.0158 (18)
C2	0.0398 (19)	0.0398 (18)	0.052 (2)	0.0141 (15)	0.0100 (16)	0.0152 (16)
C3	0.0414 (19)	0.0273 (15)	0.0457 (19)	0.0118 (13)	0.0092 (15)	0.0123 (14)
C4	0.051 (2)	0.0229 (14)	0.0346 (16)	0.0120 (13)	0.0141 (14)	0.0138 (12)
C5	0.0391 (17)	0.0250 (14)	0.0326 (16)	0.0099 (12)	0.0100 (13)	0.0109 (12)
C6	0.053 (2)	0.0245 (14)	0.0361 (17)	0.0128 (13)	0.0157 (14)	0.0141 (13)
C7	0.0378 (17)	0.0276 (14)	0.0322 (15)	0.0124 (13)	0.0100 (13)	0.0149 (12)
C8	0.0374 (17)	0.0283 (14)	0.0305 (15)	0.0098 (12)	0.0073 (12)	0.0131 (12)
C9	0.0305 (16)	0.0340 (15)	0.0302 (15)	0.0090 (13)	0.0054 (12)	0.0143 (13)
C10	0.0339 (17)	0.0366 (16)	0.0323 (16)	0.0091 (13)	0.0071 (13)	0.0164 (13)
C11	0.0414 (18)	0.0320 (15)	0.0290 (15)	0.0101 (13)	0.0057 (13)	0.0099 (13)
C12	0.0385 (18)	0.0393 (17)	0.0312 (16)	0.0139 (14)	0.0084 (13)	0.0156 (14)
C13	0.0305 (16)	0.0301 (15)	0.0334 (16)	0.0066 (12)	0.0041 (12)	0.0038 (13)
C14	0.0339 (18)	0.0309 (16)	0.0451 (19)	0.0074 (13)	0.0027 (14)	0.0066 (14)
C15	0.049 (2)	0.0377 (19)	0.069 (3)	0.0014 (16)	0.0086 (18)	0.0158 (18)
C16	0.074 (3)	0.0260 (15)	0.0331 (17)	0.0057 (15)	0.0109 (16)	0.0148 (13)
C17	0.085 (3)	0.0392 (19)	0.050 (2)	0.0057 (19)	0.026 (2)	0.0244 (17)
C18	0.096 (3)	0.0300 (17)	0.049 (2)	0.0127 (18)	0.004 (2)	0.0191 (16)
C19	0.097 (3)	0.0339 (18)	0.043 (2)	−0.0061 (19)	0.003 (2)	0.0219 (16)
C20	0.046 (2)	0.0450 (18)	0.0281 (16)	0.0178 (15)	0.0068 (14)	0.0154 (14)
C21	0.107 (4)	0.065 (3)	0.0338 (19)	0.051 (2)	0.019 (2)	0.0190 (18)
C22	0.056 (2)	0.078 (3)	0.0365 (19)	0.001 (2)	0.0018 (16)	0.0340 (19)
C23	0.048 (2)	0.0476 (19)	0.0347 (17)	0.0154 (16)	0.0007 (15)	0.0153 (15)
C24	0.043 (3)	0.038 (2)	0.034 (2)	0.014 (2)	0.0033 (19)	0.0117 (19)
C25	0.043 (3)	0.049 (3)	0.046 (2)	0.005 (2)	0.003 (2)	0.005 (2)
C26	0.058 (3)	0.063 (3)	0.040 (2)	0.013 (2)	0.001 (2)	−0.003 (2)
C27	0.073 (4)	0.060 (3)	0.037 (2)	0.025 (3)	0.008 (2)	0.011 (2)
C28	0.070 (4)	0.050 (3)	0.047 (3)	0.021 (3)	0.017 (3)	0.022 (2)
C29	0.069 (4)	0.036 (2)	0.033 (2)	0.017 (2)	0.008 (2)	0.0136 (19)
C24A	0.044 (7)	0.036 (6)	0.023 (6)	0.006 (6)	0.001 (6)	0.018 (5)
C25A	0.048 (7)	0.055 (7)	0.031 (6)	0.007 (6)	0.002 (5)	0.020 (5)
C26A	0.050 (7)	0.053 (7)	0.038 (6)	0.002 (6)	0.000 (6)	0.014 (5)
C27A	0.049 (7)	0.042 (7)	0.034 (7)	0.005 (6)	0.002 (6)	0.010 (6)
C28A	0.045 (7)	0.035 (6)	0.033 (6)	0.017 (5)	0.007 (6)	0.014 (5)
C29A	0.041 (7)	0.028 (6)	0.033 (6)	0.008 (6)	0.002 (6)	0.017 (5)
C30	0.074 (3)	0.043 (2)	0.043 (2)	−0.0019 (19)	0.0022 (19)	0.0182 (17)
C31	0.081 (3)	0.057 (2)	0.051 (2)	−0.007 (2)	−0.009 (2)	0.034 (2)
C32	0.111 (4)	0.041 (2)	0.054 (2)	−0.005 (2)	−0.024 (3)	0.0244 (19)
C33	0.116 (5)	0.056 (3)	0.060 (3)	0.016 (3)	−0.001 (3)	0.017 (2)
C34	0.103 (4)	0.062 (3)	0.085 (3)	0.024 (3)	0.029 (3)	0.024 (3)
C35	0.091 (4)	0.055 (3)	0.077 (3)	0.010 (2)	0.032 (3)	0.017 (2)
C36	0.043 (2)	0.055 (2)	0.042 (2)	−0.0004 (17)	0.0077 (16)	−0.0020 (17)
C37	0.041 (2)	0.077 (3)	0.054 (2)	0.003 (2)	0.0028 (18)	−0.009 (2)
C38	0.057 (2)	0.057 (2)	0.038 (2)	−0.0003 (19)	0.0006 (17)	0.0023 (17)
C39	0.064 (2)	0.048 (2)	0.0321 (18)	0.0109 (18)	0.0094 (16)	0.0104 (15)
C40	0.054 (2)	0.0462 (19)	0.0423 (19)	0.0122 (17)	0.0094 (16)	0.0168 (16)
C41	0.046 (2)	0.0422 (18)	0.0353 (17)	0.0000 (15)	0.0030 (14)	0.0121 (15)
O5	0.056 (2)	0.053 (2)	0.078 (4)	0.009 (2)	0.005 (2)	0.034 (2)
C42	0.074 (4)	0.050 (3)	0.055 (3)	−0.005 (3)	−0.014 (3)	0.035 (3)
C43	0.113 (5)	0.061 (3)	0.061 (3)	−0.020 (3)	−0.009 (3)	0.035 (3)
C44	0.178 (6)	0.082 (4)	0.055 (3)	−0.026 (4)	0.010 (4)	0.036 (3)
C45	0.204 (6)	0.080 (4)	0.047 (3)	−0.029 (4)	0.015 (4)	0.025 (3)
C46	0.177 (6)	0.065 (4)	0.053 (3)	−0.027 (4)	0.003 (4)	0.026 (3)
C47	0.107 (4)	0.056 (3)	0.056 (3)	−0.013 (3)	−0.004 (3)	0.029 (3)
O5A	0.058 (6)	0.052 (6)	0.027 (5)	0.000 (5)	0.003 (4)	0.021 (4)
C42A	0.046 (6)	0.062 (6)	0.020 (5)	0.012 (5)	0.003 (4)	0.020 (5)
C43A	0.037 (6)	0.072 (7)	0.028 (5)	0.004 (5)	0.001 (4)	0.029 (5)
C44A	0.041 (6)	0.088 (8)	0.043 (6)	0.002 (6)	0.006 (5)	0.026 (5)
C45A	0.050 (7)	0.101 (9)	0.045 (7)	0.000 (7)	0.015 (6)	0.013 (6)
C46A	0.054 (7)	0.091 (8)	0.048 (7)	0.007 (6)	0.004 (6)	0.005 (6)
C47A	0.047 (7)	0.073 (8)	0.037 (6)	0.015 (6)	0.012 (6)	0.001 (6)
C48	0.068 (4)	0.067 (3)	0.183 (7)	0.011 (3)	0.005 (4)	0.054 (4)
C49	0.112 (5)	0.085 (4)	0.164 (7)	0.006 (4)	0.012 (5)	0.050 (5)
C50	0.126 (6)	0.072 (4)	0.157 (7)	−0.009 (4)	0.018 (5)	0.055 (4)
C51	0.052 (2)	0.054 (2)	0.062 (2)	0.0115 (17)	0.0103 (18)	0.0383 (19)
C52	0.047 (2)	0.054 (2)	0.060 (2)	0.0032 (17)	0.0028 (17)	0.0355 (19)
C53	0.061 (2)	0.045 (2)	0.052 (2)	0.0049 (17)	0.0048 (18)	0.0283 (17)
C54	0.071 (3)	0.150 (5)	0.115 (4)	0.032 (3)	0.027 (3)	0.108 (4)

### Geometric parameters (Å, °)

**Table:**

S1—N1	1.659 (3)	C22—H22C	0.9800
S1—C3	1.711 (3)	C23—H23A	0.9800
S2—N14	1.654 (3)	C23—H23B	0.9800
S2—C13	1.725 (3)	C23—H23C	0.9800
Na1—O8	2.336 (3)	C24—C25	1.3900
Na1—N7	2.420 (3)	C24—C29	1.3900
Na1—O2	2.437 (2)	C25—C26	1.3900
Na1—N11	2.642 (3)	C25—H25	0.9500
Na1—N5	2.644 (3)	C26—C27	1.3900
O1—C8	1.253 (3)	C26—H26	0.9500
O2—C24	1.357 (3)	C27—C28	1.3900
O2—C24A	1.528 (10)	C27—H27	0.9500
O2—H2O	0.8400	C28—C29	1.3900
O3—C30	1.388 (4)	C28—H28	0.9500
O3—H3O	0.8400	C29—H29	0.9500
O4—C36	1.367 (4)	C24A—C25A	1.3900
O4—H4O	0.8400	C24A—C29A	1.3900
O6—C48	1.330 (9)	C25A—C26A	1.3900
O6—H6O	0.8400	C25A—H25A	0.9500
O7—C51	1.301 (5)	C26A—C27A	1.3900
O7—H7O	0.8400	C26A—H26A	0.9500
O8—C54	1.452 (5)	C27A—C28A	1.3900
O8—H8O	0.8400	C27A—H27A	0.9500
N1—C2	1.323 (4)	C28A—C29A	1.3900
N2—C3	1.310 (4)	C28A—H28A	0.9500
N2—C2	1.360 (4)	C29A—H29A	0.9500
N3—N4	1.295 (4)	C30—C31	1.378 (5)
N3—C3	1.389 (4)	C30—C35	1.382 (6)
N4—C4	1.351 (4)	C31—C32	1.410 (6)
N5—C6	1.314 (4)	C31—H31	0.9500
N5—N6	1.413 (3)	C32—C33	1.374 (7)
N6—C5	1.374 (4)	C32—H32	0.9500
N6—C7	1.394 (4)	C33—C34	1.367 (7)
N7—C9	1.335 (4)	C33—H33	0.9500
N7—C7	1.339 (4)	C34—C35	1.403 (6)
N8—C9	1.323 (4)	C34—H34	0.9500
N8—C8	1.363 (4)	C35—H35	0.9500
N9—C7	1.317 (4)	C36—C41	1.361 (5)
N9—C8	1.372 (4)	C36—C37	1.388 (5)
N10—C10	1.368 (4)	C37—C38	1.374 (5)
N10—C9	1.401 (4)	C37—H37	0.9500
N10—N11	1.418 (3)	C38—C39	1.369 (5)
N11—C12	1.314 (4)	C38—H38	0.9500
N12—N13	1.308 (3)	C39—C40	1.387 (5)
N12—C11	1.347 (4)	C39—H39	0.9500
N13—C13	1.378 (4)	C40—C41	1.379 (4)
N14—C14	1.309 (4)	C40—H40	0.9500
N15—C13	1.318 (4)	C41—H41	0.9500
N15—C14	1.372 (4)	O5—C42	1.358 (5)
N16—C5	1.322 (4)	O5—H5O	0.8400
N16—H16N	0.8800	C42—C43	1.3900
N16—H16M	0.8800	C42—C47	1.3900
N17—C10	1.325 (4)	C43—C44	1.3900
N17—H17N	0.8800	C43—H43	0.9500
N17—H17M	0.8800	C44—C45	1.3900
C1—C2	1.500 (5)	C44—H44	0.9500
C1—H1A	0.9800	C45—C46	1.3900
C1—H1B	0.9800	C45—H45	0.9500
C1—H1C	0.9800	C46—C47	1.3900
C4—C5	1.418 (4)	C46—H46	0.9500
C4—C6	1.438 (4)	C47—H47	0.9500
C6—C16	1.510 (4)	O5A—C42A	1.365 (12)
C10—C11	1.408 (4)	O5A—H5OA	0.8400
C11—C12	1.444 (4)	C42A—C43A	1.3900
C12—C20	1.513 (4)	C42A—C47A	1.3900
C14—C15	1.486 (5)	C43A—C44A	1.3900
C15—H15A	0.9800	C43A—H43A	0.9500
C15—H15B	0.9800	C44A—C45A	1.3900
C15—H15C	0.9800	C44A—H44A	0.9500
C16—C19	1.520 (5)	C45A—C46A	1.3900
C16—C17	1.537 (5)	C45A—H45A	0.9500
C16—C18	1.538 (4)	C46A—C47A	1.3900
C17—H17A	0.9800	C46A—H46A	0.9500
C17—H17B	0.9800	C47A—H47A	0.9500
C17—H17C	0.9800	C48—C50	1.353 (9)
C18—H18A	0.9800	C48—C49	1.374 (9)
C18—H18B	0.9800	C49—C50^i^	1.405 (8)
C18—H18C	0.9800	C49—H49	0.9500
C19—H19A	0.9800	C50—C49^i^	1.405 (8)
C19—H19B	0.9800	C50—H50	0.9500
C19—H19C	0.9800	C51—C52	1.380 (5)
C20—C23	1.521 (4)	C51—C53^ii^	1.394 (5)
C20—C22	1.528 (5)	C52—C53	1.382 (5)
C20—C21	1.531 (4)	C52—H52	0.9500
C21—H21A	0.9800	C53—C51^ii^	1.394 (5)
C21—H21B	0.9800	C53—H53	0.9500
C21—H21C	0.9800	C54—H54A	0.9800
C22—H22A	0.9800	C54—H54B	0.9800
C22—H22B	0.9800	C54—H54C	0.9800
			
N1—S1—C3	90.98 (15)	H22A—C22—H22B	109.5
N14—S2—C13	90.84 (15)	C20—C22—H22C	109.5
O8—Na1—N7	118.62 (9)	H22A—C22—H22C	109.5
O8—Na1—O2	121.65 (9)	H22B—C22—H22C	109.5
N7—Na1—O2	119.57 (9)	C20—C23—H23A	109.5
O8—Na1—N11	97.59 (9)	C20—C23—H23B	109.5
N7—Na1—N11	65.51 (8)	H23A—C23—H23B	109.5
O2—Na1—N11	109.37 (9)	C20—C23—H23C	109.5
O8—Na1—N5	104.19 (9)	H23A—C23—H23C	109.5
N7—Na1—N5	65.04 (8)	H23B—C23—H23C	109.5
O2—Na1—N5	96.00 (9)	O2—C24—C25	120.7 (3)
N11—Na1—N5	130.53 (8)	O2—C24—C29	119.3 (3)
C24—O2—C24A	15.1 (6)	C25—C24—C29	120.0
C24—O2—Na1	140.8 (2)	C24—C25—C26	120.0
C24A—O2—Na1	136.1 (5)	C24—C25—H25	120.0
C24—O2—H2O	109.5	C26—C25—H25	120.0
C24A—O2—H2O	102.3	C27—C26—C25	120.0
Na1—O2—H2O	103.0	C27—C26—H26	120.0
C30—O3—H3O	109.5	C25—C26—H26	120.0
C36—O4—H4O	109.5	C26—C27—C28	120.0
C48—O6—H6O	109.5	C26—C27—H27	120.0
C51—O7—H7O	109.5	C28—C27—H27	120.0
C54—O8—Na1	125.3 (2)	C29—C28—C27	120.0
C54—O8—H8O	109.5	C29—C28—H28	120.0
Na1—O8—H8O	113.0	C27—C28—H28	120.0
C2—N1—S1	109.0 (2)	C28—C29—C24	120.0
C3—N2—C2	108.6 (3)	C28—C29—H29	120.0
N4—N3—C3	112.2 (3)	C24—C29—H29	120.0
N3—N4—C4	114.1 (3)	C25A—C24A—C29A	120.0
C6—N5—N6	105.8 (2)	C25A—C24A—O2	115.8 (8)
C6—N5—Na1	142.0 (2)	C29A—C24A—O2	123.6 (7)
N6—N5—Na1	112.22 (17)	C24A—C25A—C26A	120.0
C5—N6—C7	127.7 (2)	C24A—C25A—H25A	120.0
C5—N6—N5	111.5 (2)	C26A—C25A—H25A	120.0
C7—N6—N5	120.7 (2)	C27A—C26A—C25A	120.0
C9—N7—C7	111.0 (2)	C27A—C26A—H26A	120.0
C9—N7—Na1	124.4 (2)	C25A—C26A—H26A	120.0
C7—N7—Na1	124.62 (19)	C28A—C27A—C26A	120.0
C9—N8—C8	115.5 (3)	C28A—C27A—H27A	120.0
C7—N9—C8	115.9 (3)	C26A—C27A—H27A	120.0
C10—N10—C9	127.2 (2)	C27A—C28A—C29A	120.0
C10—N10—N11	111.7 (2)	C27A—C28A—H28A	120.0
C9—N10—N11	120.9 (2)	C29A—C28A—H28A	120.0
C12—N11—N10	105.4 (2)	C28A—C29A—C24A	120.0
C12—N11—Na1	142.8 (2)	C28A—C29A—H29A	120.0
N10—N11—Na1	111.76 (17)	C24A—C29A—H29A	120.0
N13—N12—C11	114.4 (3)	C31—C30—C35	120.1 (4)
N12—N13—C13	111.0 (2)	C31—C30—O3	117.6 (4)
C14—N14—S2	110.1 (2)	C35—C30—O3	122.3 (4)
C13—N15—C14	108.6 (3)	C30—C31—C32	118.8 (5)
C5—N16—H16N	120.0	C30—C31—H31	120.6
C5—N16—H16M	120.0	C32—C31—H31	120.6
H16N—N16—H16M	120.0	C33—C32—C31	121.3 (4)
C10—N17—H17N	120.0	C33—C32—H32	119.3
C10—N17—H17M	120.0	C31—C32—H32	119.3
H17N—N17—H17M	120.0	C34—C33—C32	119.2 (5)
C2—C1—H1A	109.5	C34—C33—H33	120.4
C2—C1—H1B	109.5	C32—C33—H33	120.4
H1A—C1—H1B	109.5	C33—C34—C35	120.5 (5)
C2—C1—H1C	109.5	C33—C34—H34	119.8
H1A—C1—H1C	109.5	C35—C34—H34	119.8
H1B—C1—H1C	109.5	C30—C35—C34	120.0 (5)
N1—C2—N2	118.1 (3)	C30—C35—H35	120.0
N1—C2—C1	120.9 (3)	C34—C35—H35	120.0
N2—C2—C1	120.9 (3)	C41—C36—O4	122.4 (3)
N2—C3—N3	120.1 (3)	C41—C36—C37	120.2 (3)
N2—C3—S1	113.2 (2)	O4—C36—C37	117.4 (3)
N3—C3—S1	126.7 (3)	C38—C37—C36	119.9 (4)
N4—C4—C5	127.1 (3)	C38—C37—H37	120.1
N4—C4—C6	127.4 (3)	C36—C37—H37	120.1
C5—C4—C6	105.5 (3)	C39—C38—C37	120.5 (4)
N16—C5—N6	125.1 (3)	C39—C38—H38	119.8
N16—C5—C4	129.2 (3)	C37—C38—H38	119.8
N6—C5—C4	105.7 (2)	C38—C39—C40	119.0 (3)
N5—C6—C4	111.5 (3)	C38—C39—H39	120.5
N5—C6—C16	121.9 (3)	C40—C39—H39	120.5
C4—C6—C16	126.5 (3)	C41—C40—C39	120.8 (3)
N9—C7—N7	128.1 (3)	C41—C40—H40	119.6
N9—C7—N6	114.7 (3)	C39—C40—H40	119.6
N7—C7—N6	117.2 (2)	C36—C41—C40	119.5 (3)
O1—C8—N8	119.3 (3)	C36—C41—H41	120.2
O1—C8—N9	119.8 (3)	C40—C41—H41	120.2
N8—C8—N9	120.9 (3)	C42—O5—H5O	109.5
N8—C9—N7	128.5 (3)	O5—C42—C43	117.6 (3)
N8—C9—N10	114.1 (3)	O5—C42—C47	122.4 (3)
N7—C9—N10	117.4 (3)	C43—C42—C47	120.0
N17—C10—N10	126.4 (3)	C42—C43—C44	120.0
N17—C10—C11	127.6 (3)	C42—C43—H43	120.0
N10—C10—C11	105.9 (3)	C44—C43—H43	120.0
N12—C11—C10	127.9 (3)	C43—C44—C45	120.0
N12—C11—C12	126.4 (3)	C43—C44—H44	120.0
C10—C11—C12	105.7 (3)	C45—C44—H44	120.0
N11—C12—C11	111.3 (3)	C46—C45—C44	120.0
N11—C12—C20	121.8 (3)	C46—C45—H45	120.0
C11—C12—C20	126.9 (3)	C44—C45—H45	120.0
N15—C13—N13	122.4 (3)	C45—C46—C47	120.0
N15—C13—S2	112.6 (2)	C45—C46—H46	120.0
N13—C13—S2	125.0 (2)	C47—C46—H46	120.0
N14—C14—N15	117.9 (3)	C46—C47—C42	120.0
N14—C14—C15	122.0 (3)	C46—C47—H47	120.0
N15—C14—C15	120.1 (3)	C42—C47—H47	120.0
C14—C15—H15A	109.5	C42A—O5A—H5OA	109.5
C14—C15—H15B	109.5	O5A—C42A—C43A	115.2 (8)
H15A—C15—H15B	109.5	O5A—C42A—C47A	124.7 (8)
C14—C15—H15C	109.5	C43A—C42A—C47A	120.0
H15A—C15—H15C	109.5	C42A—C43A—C44A	120.0
H15B—C15—H15C	109.5	C42A—C43A—H43A	120.0
C6—C16—C19	110.0 (3)	C44A—C43A—H43A	120.0
C6—C16—C17	109.3 (3)	C43A—C44A—C45A	120.0
C19—C16—C17	110.3 (3)	C43A—C44A—H44A	120.0
C6—C16—C18	109.1 (3)	C45A—C44A—H44A	120.0
C19—C16—C18	108.6 (3)	C44A—C45A—C46A	120.0
C17—C16—C18	109.7 (3)	C44A—C45A—H45A	120.0
C16—C17—H17A	109.5	C46A—C45A—H45A	120.0
C16—C17—H17B	109.5	C47A—C46A—C45A	120.0
H17A—C17—H17B	109.5	C47A—C46A—H46A	120.0
C16—C17—H17C	109.5	C45A—C46A—H46A	120.0
H17A—C17—H17C	109.5	C46A—C47A—C42A	120.0
H17B—C17—H17C	109.5	C46A—C47A—H47A	120.0
C16—C18—H18A	109.5	C42A—C47A—H47A	120.0
C16—C18—H18B	109.5	O6—C48—C50	130.8 (7)
H18A—C18—H18B	109.5	O6—C48—C49	109.8 (8)
C16—C18—H18C	109.5	C50—C48—C49	119.1 (6)
H18A—C18—H18C	109.5	C48—C49—C50^i^	117.7 (7)
H18B—C18—H18C	109.5	C48—C49—H49	121.1
C16—C19—H19A	109.5	C50^i^—C49—H49	121.1
C16—C19—H19B	109.5	C48—C50—C49^i^	123.2 (7)
H19A—C19—H19B	109.5	C48—C50—H50	118.4
C16—C19—H19C	109.5	C49^i^—C50—H50	118.4
H19A—C19—H19C	109.5	O7—C51—C52	122.9 (4)
H19B—C19—H19C	109.5	O7—C51—C53^ii^	116.9 (4)
C12—C20—C23	109.1 (2)	C52—C51—C53^ii^	119.9 (3)
C12—C20—C22	110.1 (3)	C51—C52—C53	120.6 (4)
C23—C20—C22	108.0 (3)	C51—C52—H52	119.7
C12—C20—C21	109.8 (3)	C53—C52—H52	119.7
C23—C20—C21	108.8 (3)	C52—C53—C51^ii^	119.5 (4)
C22—C20—C21	111.0 (3)	C52—C53—H53	120.3
C20—C21—H21A	109.5	C51^ii^—C53—H53	120.3
C20—C21—H21B	109.5	O8—C54—H54A	109.5
H21A—C21—H21B	109.5	O8—C54—H54B	109.5
C20—C21—H21C	109.5	H54A—C54—H54B	109.5
H21A—C21—H21C	109.5	O8—C54—H54C	109.5
H21B—C21—H21C	109.5	H54A—C54—H54C	109.5
C20—C22—H22A	109.5	H54B—C54—H54C	109.5
C20—C22—H22B	109.5		

Symmetry codes: (i) −*x*+3, −*y*+3, −*z*+2; (ii) −*x*+1, −*y*, −*z*+1.

### Hydrogen-bond geometry (Å, °)

**Table:**

*D*—H···*A*	*D*—H	H···*A*	*D*···*A*	*D*—H···*A*
O2—H2O···O1^iii^	0.84	1.84	2.682 (2)	175
O3—H3O···N1	0.84	2.01	2.835 (3)	166
O4—H4O···N14	0.84	1.99	2.757 (3)	152
O5—H5O···N2	0.84	2.10	2.926 (6)	170
O5A—H5OA···N2	0.84	2.02	2.831 (14)	162
O7—H7O···N15	0.84	2.21	2.975 (5)	152
O8—H8O···O1^iv^	0.84	1.99	2.822 (3)	173
N16—H16M···N3	0.88	2.16	2.719 (4)	121
N16—H16N···N9	0.88	2.05	2.660 (3)	126
N17—H17M···N13	0.88	2.13	2.705 (2)	122
N17—H17N···N8	0.88	2.06	2.661 (3)	125

Symmetry codes: (iii) −*x*+2, −*y*+1, −*z*+1; (iv) −*x*+1, −*y*+1, −*z*+1.
